# A multi-trajectory analysis of commonly co-occurring mental health issues across childhood and adolescence

**DOI:** 10.1007/s00787-020-01679-1

**Published:** 2020-11-16

**Authors:** Aja L. Murray, Manuel Eisner, Daniel Nagin, Denis Ribeaud

**Affiliations:** 1 Department of Psychology, University of Edinburgh, 7 George Square, Edinburgh, EH8 9JZ UK; 2 Institute of Criminology, University of Cambridge, Cambridge, UK; 3 Jacobs Center for Productive Youth Development, University of Zurich, Zurich, Switzerland; 4 Carnegie Mellon University, Pittsburgh, PA USA

**Keywords:** Comorbidity, Developmental trajectories, Group-based trajectory modelling, Attention-deficit/hyperactivity disorder, Internalising problems, Externalising problems

## Abstract

Developmental trajectories of mental health issues can often be usefully summarised in a small number of clinically meaningful subtypes. Given the high levels of heterotypic and homotypic comorbidity in child and adolescent mental health symptoms, we explored whether it was possible to identify clinically meaningful developmental subtypes of multiple commonly co-occurring mental health issues. We evaluated the combined developmental trajectories of the most common and commonly co-occurring child and adolescent mental health issues: attention-deficit/hyperactivity disorder (ADHD), internalising, and externalising symptoms in a normative sample of youth with data (*n* = 1620) at ages 7, 8, 9, 10, 11, 12, 13 and 15 using group-based multi-trajectory modelling. Multinomial logistic regression was used to evaluate predictors of group membership. Our optimal model included six trajectory groups, labelled ‘unaffected’, ‘normative maturing’, ‘internalising’, ‘multimorbid late onset’, ‘multimorbid remitting’, and ‘multimorbid with remitting externalising’. Examining covariates of group membership suggested that males and bully victims tend to have complex mental health profiles; academic achievement and smoking during pregnancy have general associations with mental health irrespective of symptom developmental trajectories or combination; and maternal post-natal depression is primarily related to symptoms that are already in evidence by the beginning of the school years. Results suggest that developmental trajectories of commonly co-occurring mental health issues can be usefully summarised in terms of a small number of developmental subtypes. These subtypes more often than not involve multiple co-occurring mental health issues. Their association with mental health covariates depends on the combination and developmental timing of symptoms in ways that suggest they can be clinically informative.

## Introduction

There is considerable variation across individuals in mental health symptom developmental trajectories. Often this can be usefully summarised in terms of just a small number of trajectory classes that can provide a clinically useful basis for subtyping. Early work, for example, delineated two major developmental trajectories of externalising problems: life-course persistent and adolescent limited [23], incorporated into diagnostic criteria for conduct disorder as a late versus early onset specifier [5]. Analyses of trajectory groups have been similarly informative in other domains, such as ADHD and internalising problems where there is now some discussion about adopting similar developmental specifiers [28, 36]. Mental health issues, however, show a strong tendency to cluster within individuals, even for supposedly distinct domains such as externalising and internalising problems (e.g., see Beauchaine and Cicchetti [7] for an overview). As such, to illuminate the development of mental health issues and their multimorbidity, it is essential to consider the co-development of symptoms across multiple domains when modelling potential developmental subtypes.

Few studies have evaluated trajectory classes of mental health issues across multiple domains simultaneously (see [14, Girard, Tremblay, Nagin, and Côté 2019; 34, 37] for exceptions); however, the few that have provide initial demonstrations of the value of the approach. A small number of studies have, for example, used a growth mixture parallel process model approach [37, 47] to identify trajectory classes jointly defined by externalising and internalising symptoms. Using age 3–11 data from the UK-based Millennium Cohort Study, for example, Patalay et al. [37] identified 5 trajectory groups in their optimal model. These were labelled ‘low symptoms’, ‘moderate behavioural’, ‘moderate emotional’, ‘high emotional and moderate behavioural’ and ‘high behavioural and moderate emotional’. Wiggins et al. [47] used a similar technique using age 3–9 data from the US-based Fragile Families study. Their optimal model included three joint trajectories, labelled ‘normative’ (initially low and declining internalising problems with initially medium and declining externalising problems), ‘severe-decreasing’ (initially medium but decreasing internalising problems with initially high but decreasing externalising problems), and ‘severe’ (initially medium and increasing internalising problems with initially high but slightly decreasing externalising problems).

An important gap in these studies relates to the co-development of externalising and internalising problems with other common symptoms in youth. ADHD symptoms are likely to be particularly relevant for understanding how and why externalising and internalising problems co-develop. ADHD is among the most common disorders in childhood, affecting around 5–7% globally [39, Polanczyk et al. 2015; Thomas et al. 2015] and it is known to show significant comorbidity with both internalising problems [17] and externalising problems [3]. Moreover, developmental psychopathological theories suggest that, ADHD symptoms are causally antecedent to both internalising and externalising problems [8, 24; Murray et al. 2020], thus providing an important potential link between internalising and externalising trajectories,

However, describing developmental trajectory groups is primarily helpful if they map to clinically meaningful groups that, for example, differ in etiology, outcomes, or treatment responses. By extension, identifying the factors that differentiate trajectory groups can inform early identification of the symptom trajectories that a child is most likely to follow and can thus help inform early diagnosis and prediction of likely support needs and optimal treatments. However, there is currently very little information available on covariates of joint trajectory group membership, and where covariates have been examined, most fail to differentiate between groups affected by elevated symptoms but with different profiles in terms of predominant symptoms [14, Hinnant and El-Sheikh 2013, 37]. Patalay et al. [37], for example, examined predictors of the five joint emotional/behavioural problems trajectories that they identified in the Millennium Cohort Study. Candidate predictors included sex, ethnicity, income, parental education, parental occupation, lone family status, number of siblings, maternal and paternal psychological distress, parent relationship state, parent–child conflict and closeness, smoking household, maternal age at birth, unplanned pregnancy, birthweight, smoking during pregnancy, gross motor delays, relative age, child temperament dimensions; and early childhood physical health, cognitive ability, self-regulation and emotional dysregulation. However, only a small subset of predictors differentiated between children with more prominent emotional versus more prominent behavioural symptoms when overall levels of (emotional + behavioural) symptoms were similar. For example, only sex, ethnicity, maternal age at birth and infant apprehension predicted membership in the group where emotional symptoms were predominant at higher overall levels of symptoms. Similarly, only sex, ethnicity, having 2 siblings (but not 1 or 3), smoking during pregnancy, maternal psychological distress, parent–child conflict, and infant apprehension predicted membership in the groups where emotional symptoms were predominant at moderate overall levels of symptoms.

Given the lack of research to date on the joint developmental trajectories of ADHD, internalising and externalising problem symptoms, we examined joint developmental trajectories in these domains in a normative sample of youth measured at ages 7, 8, 9, 10, 11, 12, 13, and 15 in the z-proso study. We also evaluated whether established covariates of these common mental health issues in youth differentiated individuals who were assigned to the trajectory classes that emerged. There are a very large number of covariates that have been previously linked to mental health issues in childhood and adolescence, many of which were available for our sample; however, for practical reasons of alpha inflation control we limited our analyses to just a subset of candidate covariates. We selected these predictors based on seeking to cover risk factors at different stages of development and based on prior evidence of representing promising candidates for differentiating trajectories dominated by symptoms in different domains. The inclusion of covariates relating to three different stages of development was based on prior evidence that mental health developmental subtypes may correspond to the presence of risk factors and outcomes at different stages of development [36]. We thus evaluated two perinatal risk factors: maternal smoking during pregnancy and maternal post-natal depression [35, 44]; two childhood covariates: child sensation-seeking and socioeconomic status (SES) at age 7 (previous research suggests that SES in childhood is more strongly linked to mental health issues than SES in adolescence; [40]) and two early adolescence covariates: bullying victimisation and academic achievement at age 11 [4, 22]. Though difficult to identify covariate-specific associations because of mental health comorbidity and other confounding factors, past research has suggested that these predictors also show differential relations with ADHD, externalising problems, and internalising problems. Specifically, smoking during pregnancy may be particularly strongly related to ADHD and externalising problems [44]; maternal depression to internalising problems [14]; sensation-seeking to ADHD and externalising problems (e.g., [16, 19]); SES to ADHD and externalising problems [40]; bullying victimisation to internalising problems [4]; and academic achievement to ADHD and externalising problems [22, 40]. However, with only a few exceptions there has been little consideration of the relations between these covariates and combinations of mental health problems, especially taking their developmental trajectories into account. We hypothesised that smoking during pregnancy, sensation-seeking, SES, and academic achievement would differentiate any trajectory groups involving elevated ADHD and externalising problems from groups not affected by elevated symptoms in these domains, irrespective of whether these trajectories also involved internalising problems. On the other hand, we hypothesised that maternal post-natal depression and bullying victimisation would differentiate trajectories involving elevated internalising problems from those unaffected by symptoms in this domain, irrespective of whether these trajectories also involved elevated ADHD symptoms and externalising problems.

## Methods

### Ethical considerations

Ethical approval was obtained from the Ethics Committee of the Faculty of Arts and Social Sciences of the University of Zurich.

#### Participants

Participants were from the Zurich Project on Social Development from Childhood to Adulthood (z-proso) longitudinal cohort study. The current study used the teacher-reported data, which was available at waves ages 7, 8, 9, 10, 11, 12, 13, and 15, beginning in 2004. Participants were selected via a stratified random sample of schools in Zurich. First, all 90 public primary schools in the city of Zurich were blocked by size and school district, the latter to take account of area-based socio-economic variation. Next, 14 groups of schools were created crossing size and SES and four schools randomly drawn from each. All fifty-six sampled schools took part as participation was made mandatory by the school authorities. Within these schools, all children entering first grade were invited to participate, giving a target sample of 1675 from 116 classes, of whom 1620 contributed data utilised in the current study.

At baseline, most participating children (90%) were born between May 1997 and April 1998, October 1997 being the mean month of birth. Approximately half (51.9%) were male. While almost 90% of the sample were born in Switzerland, only a minority (42.6%) of their female primary caregivers and a similar proportion of their male primary caregivers were born in Switzerland. Other common primary caregiver nations of origin included Germany, Italy, Serbia and Montenegro, Yugoslavia, and Turkey. The mean International Socio-Economic Index of Occupational Status (ISEI) score [15] was 44.82 (approximately corresponding to the occupational prestige of a book-keeping clerk; SD = 17.75).

Considerable efforts were made to maximise recruitment and retention in the study. At baseline, for example, contact letters were written in the 10 languages most commonly spoken by parents, with fieldworkers who were native speakers of these languages assigned to recruit and interview parents. Incentives, translated support letters from schools, monetary incentives, and follow-up by phone were also employed to enhance participation. These measures helped achieve good response rates, with some data available for 97% of the children in the original target sample, allowing them to be included in the current analysis.

Non-response and attrition for this sample has been complex and non-monotonic due to the pattern of consent renewals at various phases and the fact that parents could decline to provide information on their child and yet still consent to teachers providing information on their child. This meant that some children have data only from a subset of informants (self-versus teacher versus parents) and/or at a subset of waves, including some cases of children who did not initially participate in the study due to a lack of parental consent but who joined the study at a later stage when consent was collected directly from participating children. The number of participants with teacher-reported mental health data (the variables used to define the trajectories in the current study) at each wave were for age 7: *n* = 1349; age 8: *n* = 1344; age 9: *n* = 1293; age 10: *n* = 1269; age 11: *n* = 1063; age 12: *n* = 976; age 13: *n* = 1268; and age 15: *n* = 1292.

Analyses of non-response suggested that the participating sample differs little from those who did not participate [13]. The main difference is that children who did not participate at baseline were more likely to have a primary caregiver who did not speak German (the official language of the study location) as their first language.

### Procedure

Self-reported questionnaire data (bullying victimisation at age 11) were collected as part of a broader questionnaire measuring psychosocial development and administered in German, the official local language, in paper and pencil format. Data were collected in groups of between 3 and 25 students in a classroom setting but during leisure time with no teacher present. Between 1 and 3 fieldworkers were present to lead the data collection sessions and provide assistance where needed. Behavioural data (sensation-seeking) were also collected from the children at age 7, the procedure for which is described in the Measures section.

Primary caregiver-reported questionnaire data (perinatal risk factors) were collected using computer assisted personal interviews (CAPI) in one of 10 languages, depending on the mother tongue of the respondent. Interviews were conducted in the home of the primary caregiver by trained fieldworks. The data used in the current study were part of a broader questionnaire assessing child psychosocial development, developmental history, and family background.

Teacher-reported data (ADHD, internalising problems, externalising problems, and academic achievement data) were collected by mail and were part of a broader questionnaire measuring child psychosocial development. The questionnaires were administered in German in paper and pencil format.

### Measures

Externalising, internalising, and ADHD symptoms were measured using an adapted teacher report version of the Social Behavior Questionnaire [45]. Within the externalising domain, 6 items measured oppositional defiant disorder and conduct disorder and 9 measured aggression. Within the internalising domain, 3 items measured anxiety and 4 measured depression. Within the ADHD domain, 4 items measured inattention and 4 measured hyperactivity/impulsivity. Inattention and hyperactivity/impulsivity were combined into a single composite because of their high correlation and similarity of developmental trajectories in z-proso [26, 28]. Composite scores were created for each SBQ subscale by item score summation. All items were identical across the measurement waves included in the current study. The reliability and validity of the SBQ scores have been supported in previous research [28, 29, 45]. In the current study the omega reliability [21] values were all > 0.90. Teacher reports were used for the mental health data because they covered the entire range of mandatory schooling (ages 7–15) in the study location in the same format. Self-reports were available for a similar age range but switched from computerised to questionnaire format in adolescence and were therefore not comparable across childhood and adolescence. They were also less comprehensive than the teacher-reports. Parent-reports were available only up until late childhood and were not available for adolescence.

Maternal smoking during pregnancy was measured using an item: ‘Did you smoke cigarettes during your pregnancy?’ administered to primary caregivers as part of the baseline assessment. Response options offered were yes, no, not applicable, don’t know/can’t remember and no answer. In some cases (*n* = 75), it was not the mother who responded to the questionnaire. In these cases, the respondent (e.g., the father) was asked whether the mother had smoked during the pregnancy.

Maternal post-natal depression was measured using an item: ‘After < child name > ’s birth did you suffer from post-natal depression?’. As with maternal smoking during pregnancy, in cases where the mother was not the informant (*n* = 75), the informant was asked whether the mother experienced post-natal depression.

Sensation-seeking at age 7 was measured using an adapted 9-item version of the travel game developed by Alsaker and Gutzwiller-Helfenfinger [2], comprehensively described in Murray, Eisner, Obsuth et al. [28]. In brief, scores were derived from a behavioural game ‘The Travel Game’ in which children could choose different options that were more or less ‘sensation-seeking’. Assessments were carried out individually by specially trained investigators and took place during normal school time. Omega reliability for the scale in the current sample was 0.80. Composite scores were derived by summation of the individual item scores.

Bullying victimisation at age 11 was measured using the self-reported 4-item Zurich Brief Bullying Scales (ZBBS; [25]). The ZBBS as was administered at the age 11 wave of z-proso includes four victimisation items referring to being purposely ignored or excluded; laughed at, mocked or insulted; hit, bitten, kicked or having hair pulled; and having possessions stolen, broken or hidden. The items were self-reported and measured frequency of victimization on a six-point scale from never to (almost) every day. Omega reliability for the ZBBS victimization items in the current sample was 0.72. Composite scores were derived by summation of the individual item scores.

Academic achievement at age 11 was measured as the average of maths and language competence scores. These scores were provided by teachers based who rated the child’s competence in each domain on a five-point scale from much worse to much better [than the average student]. The correlation between maths and language competence scores was *r* = 0.72 (*p* < 0.001).

### Statistical procedure

To explore whether we could parse the heterogeneity in joint ADHD, externalising, and internalising trajectories into meaningful subgroups, we used group based multi-trajectory analysis, comprehensively described in [32]. In brief, GBTM is a form of finite mixture modelling for longitudinal data and group based multi-trajectory modelling provides a generalisation of the technique to situations where trajectory group membership may be defined by multiple indicators. Unlike growth mixture modelling, it does not permit within-class variation, reflecting the fact that the classes are conceptualised as a convenient summary of a continuous distribution rather than representing true subtypes. We fit models with between 1 and 6 classes and compared the Akaike’s information criterion (AIC), Bayesian information criterion (BIC) and sample size adjusted BIC (saBIC) associated with each for the purposes of model selection. We did not go beyond 6 classes in order to preserve parsimony given the sample size available. Models with linear growth only and models with both linear and quadratic growth were fit. Given how AIC, BIC and saBIC values are calculated for these models, larger (more positive) values indicate better fitting models in this context [30]. These models were fit using Stata version 15.

We then examined the association between covariates of common mental health issues and class membership based on our chosen ‘best fitting’ model. Class membership was regressed on the covariates in a series of multinomial logistic regressions, in a single step. In contrast to other approaches to modelling heterogeneity in longitudinal trajectories (see e.g., Asparouhov and Muthén 2014), it has been shown the inclusion of predictors is unlikely to affect the formation of groups in GBTM, therefore, multi-step methods are not necessary [41]. To help ensure this we used the parameter estimates from the models without any predictors as the starting values for the trajectory parameters in the model with the predictors and subsequently checked that the model-predicted values did not differ substantively across the models with and without predictors. Missing data were dealt with using multivariate imputation with chained equations, using the mice package in R [9]. The imputation model included all of the previously described covariates, variables previously identified as predictors of attrition in this sample [13], ADHD, externalising, and internalising, and several putative outcome variables discussed in a related paper (delinquency, social exclusion, optimism, intimate partner violence perpetration and victimisation; [25]). We used three imputed datasets, with results pooled using Rubin’s rules [43]. We used an imputation approach rather than a weighting approach to deal with non-random attrition because this allowed us to include more datapoints, especially given that attrition was non-monotonic and involved item- as well as unit non-response (e.g., Seaman et al. 2012). This method yields unbiased parameter estimates provided that data are missing at random (MAR; [42]).

## Results

**Fig. 1:**
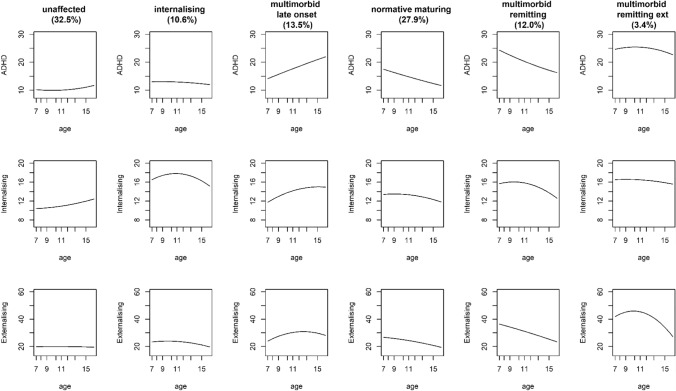
Trajectories for the ‘best-fitting’ (6-group) model based on imputation 1

**Fig. 2:**
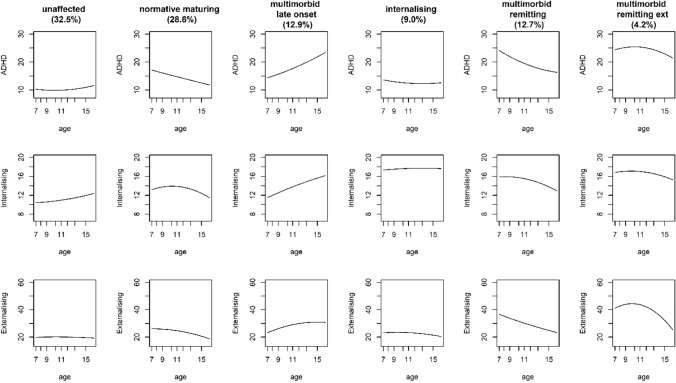
Trajectories for the ‘best-fitting’ (6-group) model based on imputation 2

**Fig. 3:**
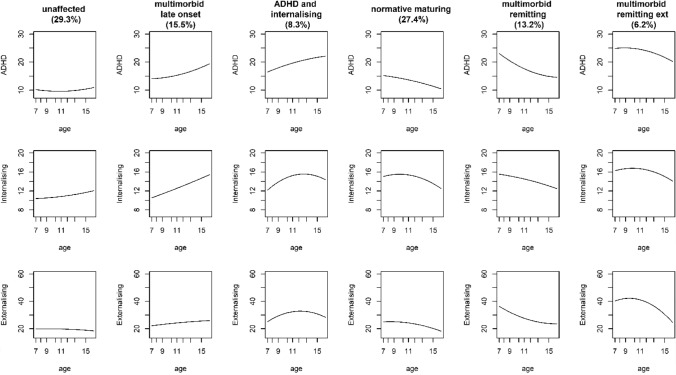
Trajectories for the ‘best-fitting’ (6-group) model based on imputation 3

Descriptive statistics are provided in Table 1. Before interpreting the pooled results, models from the three imputations were inspected and are presented separately for each imputation in order to ensure that the same GBTM model emerged across the imputations. Fit statistics across the three imputed datasets are provided in Table 2. Fit statistics mainly favoured the 6-group model with quadratic growth, though BIC (which has the larger parsimony penalty) sometimes favoured the 6-group model with linear growth only. On balance, we preferred the model with both linear and quadratic growth because it allowed us to avoid the possibility of mis-specifying non-linear growth as linear. Figure 1 summarises this model, based on the parameter estimates from the first imputation (parameter estimates from all imputations were highly similar and are provided in Tables 3,4,5 and plotted in Figs. 2 and 3).

**Table 1:** Descriptive statistics

	n	Mean	SD
ADHD age 7	1312	15.66	7.04
ADHD age 8	1305	14.59	6.91
ADHD age 9	1283	14.34	6.69
ADHD age 10	1252	14.59	6.96
ADHD age 11	1053	14.31	6.97
ADHD age 12	970	13.78	6.62
ADHD age 13	1242	14.08	6.69
ADHD age 15	1276	13.92	6.56
Internalising age 7	1302	13.03	5.29
Internalising age 8	1303	12.43	5.08
Internalising age 9	1281	12.90	5.19
Internalising age 10	1240	13.24	5.15
Internalising age 11	1034	13.31	5.32
Internalising age 12	967	13.19	5.39
Internalising age 13	1232	13.13	5.33
Internalising age 15	1265	13.09	5.27
Externalising age 7	1263	25.08	9.62
Externalising age 8	1266	24.67	9.08
Externalising age 9	1240	25.20	9.45
Externalising age 10	1213	24.28	9.48
Externalising age 11	1039	23.75	8.78
Externalising age 12	953	23.97	9.35
Externalising age 13	1207	22.28	8.05
Externalising age 15	1221	22.29	7.79
Gender	Male = 870; female = 805		
Smoking during pregnancy	Exposed = 197; not exposed = 1023		
Maternal post-natal depression	Exposed = 160; not exposed = 1058		
Sensation seeking age 7	1355	13.95	1.35
Bully victimisation age 11	1140	7.11	3.16
Academic achievement age 11	1061	6.56	2.37

**Table 2:** Model fit statistics for GBTM models of up to 6 groups across the three imputed datasets

Model	Imputation 1				Imputation 2				Imputation 3			
	BIC_N	BIC_n	AIC	LL	BIC_N	BIC_n	AIC	LL	BIC_N	BIC_n	AIC	LL
1L	− 128,434	− 128,420	− 128,396	− 128,387	− 128,521	− 128,507	− 128,483	− 128,474	− 128,492	− 128,478	− 128,454	− 128,445
1Q	− 128,421	− 128,402	− 128,370	− 128,358	− 128,509	− 128,490	− 128,458	− 128,446	− 128,481	− 128,462	− 128,429	− 128,417
2L	− 124,951	− 124,926	− 124,882	− 124,866	− 125,041	− 125,016	− 124,973	− 124,957	− 124,976	− 124,950	− 124,907	− 124,891
2Q	− 124,938	− 124,903	− 124,843	− 124,821	− 125,032	− 124,998	− 124,938	− 124,916	− 124,963	− 124,928	− 124,869	− 124,847
3L	− 123,695	− 123,659	− 123,597	− 123,574	− 123,803	− 123,766	− 123,704	− 123,681	− 123,768	− 123,731	− 123,669	− 123,646
3Q	− 123,681	− 123,630	− 123,544	− 123,512	− 123,792	− 123,741	− 123,655	− 123,623	− 123,750	− 123,699	− 123,613	− 123,581
4L	− 123,223	− 123,175	− 123,094	− 123,064	− 123,435	− 123,388	− 123,307	− 123,277	− 123,368	− 123,320	− 123,239	− 123,209
4Q	− 123,213	− 123,146	− 123,033	− 122,991	− 123,314	− 123,247	− 123,134	− 123,092	− 123,307	− 123,240	− 123,127	− 123,085
5L	− 122,867	− 122,808	− 122,708	− 122,671	− 122,939	− 122,880	− 122,781	− 122,744	− 122,943	− 122,884	− 122,785	− 122,748
5Q	− 122,854	− 122,771	− 122,631	− 122,579	− 122,931	− 122,849	− 122,709	− 122,657	− 122,925	− 122,842	− 122,702	− 122,650
6L	− 122,668	− 122,598	− 122,479	− 122,435	− **122,761**	− 122,691	− 122,573	− 122,529	− **122,728**	− **122,658**	− 122,539	− 122,495
6Q	**− 122,667**	− **122,569**	− **122,402**	− **122,340**	− 122,767	− **122,668**	− **122,501**	− **122,439**	− 122,776	− 122,678	− **122,511**	− **122,449**

Best fit value across models compared in bold

*L* linear only, *Q* linear and quadratic, *BIC_N* Bayesian information criterion adjusted for sample size, *BIC_n* BIC adjusted for number of observations, *AIC* Akaike information criterion, *LL* log-likelihood

**Table 3:** Trajectory parameter estimates from ‘best fitting’ (6-class with linear and quadratic growth) model in first imputed dataset

Group	Parameter	Estimate	SE	*T*	*P*
ADHD					
1	Intercept	13.757	1.443	9.536	< 0.001
	Linear	− 0.760	0.262	− 2.901	0.004
	Quadratic	0.038	0.011	3.295	0.001
2	Intercept	11.766	3.059	3.847	< 0.001
	Linear	0.286	0.559	0.512	0.609
	Quadratic	− 0.016	0.024	− 0.661	0.508
3	Intercept	7.050	2.595	2.716	0.007
	Linear	1.004	0.472	2.129	0.033
	Quadratic	− 0.008	0.021	− 0.365	0.715
4	Intercept	23.033	2.086	11.042	< 0.001
	Linear	− 0.800	0.361	− 2.213	0.027
	Quadratic	0.008	0.015	0.502	0.615
5	Intercept	33.567	2.619	12.816	< 0.001
	Linear	− 1.410	0.476	− 2.964	0.003
	Quadratic	0.023	0.020	1.133	0.257
6	Intercept	17.168	4.565	3.761	< 0.001
	Linear	1.533	0.826	1.856	0.064
	Quadratic	− 0.071	0.036	− 1.981	0.048
Internalising					
1	Intercept	10.874	1.398	7.779	< 0.001
	Linear	− 0.183	0.253	− 0.724	0.469
	Quadratic	0.016	0.011	1.474	0.141
2	Intercept	6.913	3.444	2.008	0.045
	Linear	1.916	0.597	3.208	0.001
	Quadratic	− 0.084	0.025	− 3.331	0.001
3	Intercept	3.458	2.421	1.429	0.153
	Linear	1.456	0.440	3.311	0.001
	Quadratic	− 0.046	0.019	− 2.400	0.016
4	Intercept	11.094	1.801	6.160	< 0.001
	Linear	0.519	0.325	1.594	0.111
	Quadratic	− 0.028	0.014	− 1.989	0.047
5	Intercept	9.528	2.370	4.020	< 0.001
	Linear	1.325	0.426	3.114	0.002
	Quadratic	− 0.067	0.018	− 3.667	< 0.001
6	Intercept	15.184	4.395	3.455	0.001
	Linear	0.301	0.794	0.379	0.705
	Quadratic	− 0.016	0.034	− 0.472	0.637
Externalising					
1	Intercept	18.695	2.036	9.181	< 0.001
	Linear	0.235	0.371	0.634	0.526
	Quadratic	− 0.011	0.016	− 0.681	0.496
2	Intercept	15.101	4.288	3.522	< 0.001
	Linear	1.759	0.790	2.226	0.026
	Quadratic	− 0.088	0.034	− 2.538	0.011
3	Intercept	− 4.695	3.785	− 1.240	0.215
	Linear	5.340	0.694	7.697	< 0.001
	Quadratic	− 0.200	0.030	− 6.605	< 0.001
4	Intercept	27.843	2.927	9.512	< 0.001
	Linear	0.124	0.522	0.238	0.812
	Quadratic	− 0.037	0.022	− 1.638	0.102
5	Intercept	44.455	4.030	11.031	< 0.001
	Linear	− 0.946	0.727	− 1.302	0.193
	Quadratic	− 0.017	0.031	− 0.559	0.577
6	Intercept	− 2.751	6.445	− 0.427	0.670
	Linear	9.286	1.161	8.001	< 0.001
	Quadratic	− 0.443	0.050	− 8.809	< 0.001

Group 1 = unaffected (*n* = 527; 32.5% of sample); group 2 = internalising (*n* = 172; 10.6%); group 3 = multimorbid late onset (*n* = 219; 13.5%); group 4 = normative maturing (*n* = 452; 27.8%); group 5 = multimorbid remitting (*n* = 195; 21%) g; group 6 = multimorbid externalising remitting (*n* = 55; 3.4%)

**Table 4:** Trajectory parameter estimates from ‘best fitting’ (6-class with linear and quadratic growth) model in second imputed dataset

Group	Parameter	Estimate	SE	*t*	*p*
ADHD					
1	Intercept	14.420	1.445	9.978	< 0.001
	Linear	− 0.864	0.261	− 3.303	0.001
	Quadratic	0.041	0.011	3.626	< 0.001
2	Intercept	21.625	1.993	10.853	< 0.001
	Linear	− 0.627	0.347	− 1.806	0.071
	Quadratic	0.003	0.015	0.203	0.839
3	Intercept	10.746	2.759	3.895	< 0.001
	Linear	0.285	0.506	0.563	0.574
	Quadratic	0.027	0.022	1.199	0.230
4	Intercept	18.484	3.325	5.558	< 0.001
	Linear	− 0.892	0.586	− 1.522	0.128
	Quadratic	0.032	0.025	1.288	0.198
5	Intercept	37.024	2.551	14.516	< 0.001
	Linear	− 2.136	0.467	− 4.574	< 0.001
	Quadratic	0.054	0.020	2.670	0.008
6	Intercept	13.863	4.143	3.346	0.001
	Linear	2.162	0.749	2.886	0.004
	Quadratic	− 0.101	0.032	− 3.103	0.002
Internalising					
1	Intercept	10.672	1.401	7.617	< 0.001
	Linear	− 0.138	0.254	− 0.541	0.588
	Quadratic	0.014	0.011	1.258	0.208
2	Intercept	6.424	1.929	3.330	0.001
	Linear	1.384	0.338	4.098	< 0.001
	Quadratic	− 0.064	0.014	− 4.508	< 0.001
3	Intercept	6.497	2.512	2.587	0.010
	Linear	0.755	0.457	1.651	0.099
	Quadratic	− 0.011	0.020	− 0.559	0.576
4	Intercept	15.956	4.221	3.780	< 0.001
	Linear	0.251	0.723	0.347	0.728
	Quadratic	− 0.009	0.030	− 0.298	0.766
5	Intercept	12.831	2.357	5.445	< 0.001
	Linear	0.719	0.423	1.702	0.089
	Quadratic	− 0.042	0.018	− 2.282	0.023
6	Intercept	13.082	3.916	3.341	0.001
	Linear	0.792	0.705	1.122	0.262
	Quadratic	− 0.039	0.031	− 1.290	.197
Externalising					
1	Intercept	17.649	2.051	8.606	< 0.001
	Linear	0.451	0.374	1.206	0.228
	Quadratic	− 0.021	0.016	− 1.301	0.193
2	Intercept	21.164	2.697	7.847	< 0.001
	Linear	1.267	0.477	2.655	0.008
	Quadratic	− 0.083	0.020	− 4.077	< 0.001
3	Intercept	2.687	3.879	0.693	0.488
	Linear	3.620	0.714	5.073	< 0.001
	Quadratic	− 0.116	0.031	− 3.688	< 0.001
4	Intercept	18.018	4.618	3.902	< 0.001
	Linear	1.110	0.838	1.325	0.185
	Quadratic	− 0.057	0.036	− 1.573	0.116
5	Intercept	50.683	3.982	12.728	< 0.001
	Linear	− 2.100	0.717	− 2.930	0.003
	Quadratic	0.029	0.031	0.947	0.344
6	Intercept	0.050	6.156	0.008	0.994
	Linear	8.636	1.101	7.845	< 0.001
	Quadratic	− 0.420	0.047	− 8.917	< 0.001

Group 1 = unaffected (*n* = 528; 32.6% of sample); group 2 = normative maturing (*n* = 464; 28.6%); group 3 = multimorbid late onset (*n* = 210; 12.9%); group 4 = internalising (*n* = 146; 9%); group 5 = multimorbid remitting (*n* = 205; 12.7%); group 6 = multimorbid with remitting externalising (*n* = 67; 4.2%)

**Table 5:** Trajectory parameter estimates from ‘best fitting’ (6-class with linear and quadratic growth) model in third imputed dataset

Group	Parameter	Estimate	SE	*t*	*p*
ADHD					
1	Intercept	14.893	1.537	9.687	< 0.001
	Linear	− 0.947	0.279	− 3.395	0.001
	Quadratic	0.042	0.012	3.499	0.001
2	Intercept	15.954	3.155	5.057	< 0.001
	Linear	− 0.614	0.584	− 1.051	0.293
	Quadratic	0.048	0.027	1.822	0.069
3	Intercept	8.022	3.621	2.215	0.027
	Linear	1.373	0.677	2.027	0.043
	Quadratic	− 0.032	0.031	− 1.047	0.295
4	Intercept	15.954	1.964	8.121	< 0.001
	Linear	0.068	0.348	0.195	0.846
	Quadratic	− 0.023	0.015	− 1.544	0.123
5	Intercept	39.613	2.682	14.770	< 0.001
	Linear	− 2.800	0.479	− 5.846	< 0.001
	Quadratic	0.078	0.021	3.792	< 0.001
6	Intercept	18.881	3.378	5.589	< 0.001
	Linear	1.370	0.610	2.246	0.025
	Quadratic	− 0.076	0.026	− 2.884	0.004
Internalising					
1	Intercept	10.744	1.511	7.110	< 0.001
	Linear	− 0.144	0.273	− 0.528	0.597
	Quadratic	0.013	0.012	1.086	0.277
2	Intercept	7.381	2.776	2.659	0.008
	Linear	0.388	0.519	0.748	0.455
	Quadratic	0.005	0.023	0.229	0.819
3	Intercept	− 1.431	3.529	− 0.405	0.685
	Linear	2.528	0.654	3.863	< .001
	Quadratic	− 0.094	0.029	− 3.275	0.001
4	Intercept	8.936	1.860	4.803	< 0.001
	Linear	1.298	0.326	3.984	< 0.001
	Quadratic	− 0.064	0.014	− 4.570	< 0.001
5	Intercept	16.144	2.538	6.360	< 0.001
	Linear	0.023	0.453	0.052	0.959
	Quadratic	− 0.014	0.019	− 0.725	0.469
6	Intercept	10.241	3.434	2.982	0.003
	Linear	1.263	0.617	2.046	0.041
	Quadratic	− 0.061	0.027	− 2.295	0.022
Externalising					
1	Intercept	17.472	2.137	8.174	< 0.001
	Linear	0.499	0.388	1.285	0.199
	Quadratic	− 0.026	0.017	− 1.549	0.121
2	Intercept	16.822	4.046	4.158	< 0.001
	Linear	0.858	0.780	1.100	0.271
	Quadratic	− 0.019	0.036	− 0.521	0.602
3	Intercept	− 10.430	5.568	− 1.873	0.061
	Linear	6.722	1.063	6.326	< 0.001
	Quadratic	− 0.261	0.048	− 5.445	< 0.001
4	Intercept	18.023	2.700	6.674	< 0.001
	Linear	1.661	0.481	3.456	0.001
	Quadratic	− 0.097	0.021	− 4.739	< 0.001
5	Intercept	64.262	4.765	13.485	< 0.001
	Linear	− 4.791	0.817	− 5.867	< 0.001
	Quadratic	0.141	0.034	4.144	< 0.001
6	Intercept	9.217	5.883	1.567	0.117
	Linear	6.718	1.054	6.375	< 0.001
	Quadratic	− 0.342	0.045	− 7.657	< 0.001

Group 1 = unaffected (*n* = 474; 29.3% of sample); group 2 = (*n* = 251; 15.5%); group 3 = (*n* = 135; 8.3%); group 4 = (*n* = 445; 27.4%); group 5 = (*n* = 215; 13.3%); group 6 = (*n* = 101; 6.2%)

Based on the first imputation, Group 1 (32.5% of the sample) was characterised by low levels of all three mental health issues and was, therefore, labelled ‘unaffected’. Group 2 (10.6%) was characterised by low levels of ADHD and externalising problems but elevated internalising problems and was, therefore, labelled ‘internalising’. In the third imputed dataset, this group also showed some ADHD symptom elevations, possibly reflecting the negative impact of internalising symptoms on concentration. This was the only substantive difference in the groups across the three imputations. Group 3 (13.5%) was characterised by increasing levels of ADHD, externalising problems and internalising problems over the course of development and was, therefore, labelled ‘multimorbid late onset’. Group 4 (27.9%) was characterised by initially slightly elevated levels of ADHD, externalising problems and internalising problems that declined over the course of development. As many children can show initial mild symptoms that they ‘grow out of’ (especially hyperactive and externalising problems), group 4 was labelled ‘normative maturing’. Group 5 (12.0%) was characterised by initially elevated ADHD, internalising and externalising symptoms that declined towards later adolescence. This group was, therefore, labelled ‘multimorbid remitting’. Finally, group 6 (3.4%) was characterised by stably elevated levels of ADHD and internalising symptoms but declining levels of externalising problems. Group 6 was, therefore, labelled ‘multimorbid with remitting externalising.’

### Covariates of trajectory classes

Results of the multinomial logistic regressions predicting class membership are provided in Table 6. Coefficients represent the differences between each class and the reference ‘unaffected’ class. Males were over-represented in the multimorbid late onset, multimorbid remitting, and multimorbid with remitting externalising groups but there were no gender differences in the internalising nor normative maturing groups. In terms of perinatal factors, smoking during pregnancy predicted increased risk of membership in all groups relative to the unaffected group, while maternal post-natal depression was associated with an increased risk of membership in the internalising, normative maturing, and multimorbid remitting groups only. In terms of covariates in childhood and adolescence, sensation-seeking was unrelated to membership in any of the groups; bullying victimisation predicted an increased risk of membership in all but the internalising group; and low academic achievement predicted an increased risk of membership in all groups relative to the unaffected group.

**Table 6:** Multinomial logistic regression results

Group	B	SE	Lower	Upper	OR
Gender (1 = male, 2 = female)					
Internalising	− 0.71	1.68	− 4.00	2.58	0.49
Multimorbid late onset	− 1.37*	0.56	− 2.47	− 0.27	0.25
Normative maturing	− 0.63	0.38	− 1.38	0.12	0.53
Multimorbid remitting	− 1.39*	0.22	− 1.83	− 0.95	0.25
Multimorbid with remitting externalising	− 2.13*	0.36	− 2.84	− 1.42	0.12
Smoking during pregnancy (1 = yes, 2 = no)					
Internalising	− 1.18*	0.40	− 1.96	− 0.40	0.31
Multimorbid late onset	− 1.20*	0.43	− 2.04	− 0.36	0.30
Normative maturing	− 0.87*	0.30	− 1.47	− 0.28	0.42
Multimorbid remitting	− 0.83*	0.27	− 1.36	− 0.30	0.44
Multimorbid with remitting externalising	− 1.19*	0.31	− 1.80	− 0.58	0.30
Maternal post-natal depression (1 = yes, 2 = no)					
Internalising	− 0.97*	0.34	− 1.64	− 0.29	0.38
Multimorbid late onset	− 0.64	0.48	− 1.57	0.29	0.53
Normative maturing	− 1.12*	0.46	− 2.01	− 0.22	0.33
Multimorbid remitting	− 0.88*	0.27	− 1.41	− 0.35	0.41
Multimorbid with remitting externalising	− 0.49	0.55	− 1.56	0.58	0.62
Sensation-seeking (age 7)					
Internalising	− 0.05	0.08	− 0.21	0.10	0.95
Multimorbid late onset	− 0.05	0.12	− 0.29	0.19	0.96
Normative maturing	0.01	0.06	− 0.11	0.13	1.01
Multimorbid remitting	− 0.09	0.08	− 0.24	0.06	0.92
Multimorbid with remitting externalising	0.00	0.10	− 0.20	0.19	1.00
Bullying victimisation (age 11)					
Internalising	0.06	0.04	− 0.03	0.14	1.06
Multimorbid late onset	0.14*	0.03	0.09	0.20	1.15
Normative maturing	0.09*	0.03	0.04	0.15	1.10
Multimorbid remitting	0.14*	0.03	0.08	0.20	1.15
Multimorbid with remitting externalising	0.13*	0.04	0.06	0.20	1.14
Academic achievement (age 11)					
Internalising	− 0.39*	0.09	− 0.56	− 0.23	0.67
Multimorbid late onset	− 0.41*	0.05	− 0.50	− 0.32	0.66
Normative maturing	− 0.38*	0.09	− 0.56	− 0.20	0.68
Multimorbid remitting	− 0.47*	0.06	− 0.59	− 0.35	0.62
Multimorbid with remitting externalising	− 0.52*	0.09	− 0.70	− 0.34	0.60

* significant at *p* < .05

## Discussion

In this study, we aimed to distil the combined developmental trajectories of multiple commonly co-occurring mental health issues (ADHD, internalising problems and externalising problems) into a small number of clinically meaningful trajectory groups that could be distinguished on the basis of established correlates of child and adolescent psychopathology. Using group-based trajectory modelling, we identified six trajectory groups. Two covariates: smoking during pregnancy and low academic achievement were related to membership in all groups relative to the unaffected group while others exhibited more specific associations with trajectory groups.

Two groups characterised by relatively low symptom levels and labelled ‘unaffected’ and ‘normative maturing’ respectively accounted for the majority of the sample. The former was characterised by consistently low levels of psychopathology across development while the latter showed early minor elevations only. The normative maturing group was assumed to reflect the fact that many symptoms that appear early in life, especially hyperactivity and behavioural problems disappear naturally as children’s emotional and behavioural regulation abilities improve with maturation (e.g., Lahey et al. [18]).

The remaining groups were characterised by some form of elevation of psychopathology. One group (approximately 10% of the sample, labelled ‘internalising’) was characterised by elevations primarily in internalising problems. All other groups showed elevations in multiple areas, supporting the idea that most individuals with mental health issues experience symptoms in more than one domain [33]. The developmental coupling of symptoms is not surprising in the context of contemporary models of ADHD-internalising-externalising comorbidity. These variously argue that ADHD symptoms and externalising problems can lead to anxiety and depression via associated psychosocial difficulties; that anxiety and depression may interfere with attention, exacerbating ADHD symptoms; and that ADHD symptoms may lead to externalising problems via an escalating cascade of behaviour problems [8, 17, Murray et al. 2020; 46; Wolff and Ollendick 2006].

One of the multimorbid groups (approximately 14% of the sample; labelled ‘multimorbid late onset’) was characterised by initially low but increasing in all three symptom areas across development. Another group (approximately 12% of the sample; labelled ‘multimorbid remitting’) was characterised by initially high levels of all three symptom areas that decreased over the course of development leaving some residual symptom elevation at age 15. The final group (approximately 3% of the sample; labelled ‘multimorbid with remitting externalising’) was characterised by consistently elevated ADHD and internalising symptoms but late-declining externalising problems. The presence of this group implies a need to avoid assuming that the resolution of behavioural issues (which are often the symptoms most easily detected) implies a resolution of all symptoms. Some with remitting behavioural symptom may retain high levels of internal distress and ADHD symptoms that could interfere with their functioning, as suggested by the fact that this group had poorer academic achievement and higher levels of bullying victimisation compared to the unaffected group.

Further insights into the nature of the groups were provided by comparisons of the ‘unaffected’ group with the remaining five groups. These comparisons underlined the importance of a developmental perspective that takes into account the joint trajectories of commonly co-occurring mental health issues. For example, analyses suggested that males were more likely to have complex profiles involving both behavioural and emotional difficulties. They were over-represented in the multimorbid late onset, multimorbid remitting, and multimorbid with remitting externalising groups, but not the ‘pure’ internalising group. Previous discussions have tended to focus on sex differences in emotional versus behavioural symptoms [20] and little considered their combination. However, our results suggest that males who present with behavioural problems and ADHD are likely to be experiencing co-occurring internalising problems, underlining the importance of the inclusion of these symptoms in assessments even when they are not the reason for referral.

Similarly, we found that bullying victimisation was related to groups with mixed emotional-behavioural problem profiles but not to the group with the pure internalising profile. Thus, while internalising has been associated with bullying victimisation [4], our analyses suggest that this risk could be particularly important in the context of co-occurring ADHD and behavioural problems. This is consistent with the idea that children and adolescents who have behavioural problems are liable to elicit negative reactions from their peers, leading to rejection and victimisation [11].

The importance of considering the developmental timing of symptoms was highlighted by our finding that maternal post-natal depression was associated with an increased risk of membership in groups which had early emerging symptom elevations (internalising, normative maturing, multimorbid remitting) but not the group that showed late-emerging symptoms (multimorbid late onset). Our analyses thus suggest that early exposure to maternal post-natal depression does not necessarily result in lasting symptoms, for example, in the case of the normative maturing group; nor can it account for late onset symptoms, which may be more likely to have their origins in risk factors deriving from the late childhood and early adolescent period (e.g., Parkes et al. [36]).

The fact that the groups identified were differentiable on the basis of some established risk factors for mental health issues suggests possible clinically meaningful distinctions between the groups. This merits further exploration as differences in clinically important factors such as etiology, sequelae, and treatment responses would make subtyping on the basis of trajectory groups useful for understanding the causes, support needs and optimal treatments for individuals presenting with different developmental patterns of (co-occurring) symptoms. At present, developmental trajectories are taken into account only in a small number of disorders, including conduct disorder, which has a specifier for age of onset (with an earlier age of onset indicating greater severity) [5, 26]. To the extent that the trajectory groups in the current study are replicable and show to be distinguishable on the basis of clinically meaningful factors in future studies, it could be useful for clinical diagnostic criteria to incorporate specifiers for joint developmental trajectories of multiple symptoms to efficiently encode information regarding likely etiology, outcomes, and promising interventions.

Unfortunately, the present study is among only a few to model joint mental health trajectories, and the only (to the best of our knowledge) to model joint ADHD-externalising-internalising trajectories across the school years age range. As such, there is currently little previous evidence on the extent to which the same trajectory groups emerge in different samples and can be differentiated on the basis of similar covariates to those studied here However, our results are consistent with previous studies in showing that individuals who belong to trajectory groups characterised by elevated externalising problems also tend to belong to trajectory groups characterised by elevated internalising problems (e.g., [34, 37]). Our study, however, differed in its findings from one of the few studies that explored trajectory groups jointly characterised by internalising and externalising problems in showing evidence of a ‘pure’ internalising trajectory group. Specifically, Patalay et al. [37], who examined trajectory groups in a large representative sample, found no evidence of internalising problems occurring in the absence of externalising problems, as internalising symptoms were always accompanied by externalising problems at a higher or lower severity. Our study was, on the other hand, consistent with this previous study in finding that while a number of risk factors can differentiate those who are unaffected from those affected at some point in their development by some combination of symptoms, few are specific to particular trajectory groups [37].

Our group-based trajectory modelling approach provides complementary evidence to alternative approaches to modelling the development of co-occurring mental health issues. Previous work in this and other samples have, for example, examined the extent and longitudinal evolution of ‘general comorbidity’ sometimes also referred to as the ‘*p-*factor’, finding that there is considerable co-occurrence between symptoms in different domains across childhood and adolescent development [10, 24, 25]. Our finding here that most individuals who are affected by elevated symptoms fall into trajectory groups characterised by symptoms in multiple domains is thus consistent with this previous work but also helps to identify the specific developmental course that the co-occurring symptoms take. Future research connecting these alternative approaches e.g., through modelling the developmental trajectories of higher-order general factors of psychopathology may provide further insights into the developmental dynamics of co-occurring mental health issues.

### Limitations

It is important to consider the limitations of the current study. First, the need to maintain adequate statistical power for our group comparisons limited the number of groups that could be extracted in our GBTM. Limiting our number of groups to six gave us a smallest group size that likely meant that our analyses were under-powered to detect very small effects involving this group. Such small effects were, however, judged to be unlikely to be of a magnitude where they would be clinically important. Second, we used only teacher reports of symptoms to construct our mental health trajectories. This allowed us to avoid common rater bias [38] when assessing the relations between trajectories and covariates (which were based on parent reports and youth self-reports); however, previous evidence suggests young people show different symptoms in different contexts and/or in interaction with different informants [12, 27]. This makes it important to assess the generalisability of conclusions across reports from different informants. Teacher-reports may also have some disadvantages compared with reports from other informants, especially in adolescence where their interactions with the young person may be limited. Further, though this issue is not limited to teacher-reports, teacher-reports have previously been shown to be biased by factors as halo effects [1]. Third, it was not possible to tell why improvements and deteriorations in symptoms occurred. We did not have sufficient information, for example, to evaluate the role of exposure to diagnosis and clinical interventions on symptom improvements among those showing symptom decreases over development. Group-based trajectory modelling in cohorts with more detailed information on intervention exposure and timing would help clarify the extent to which improvements are spontaneous versus attributable to treatments for mental health symptoms. Fourth, in common with all modelling approaches, it is important to consider what can and cannot be inferred from applications of the model (see [6, 31] for discussions). In particular, while GBTM seeks to provide a useful and potentially clinically meaningful summary of heterogeneous trajectories, the groups that emerge should not be taken to literally exist. Under different modelling decisions (e.g., inclusion of within-group random effects, inclusion of additional or fewer higher-order growth parameters) different groups from those that emerged in the current analysis may have been indicated and these modelling decisions, as well as the interpretation of the groups are inevitably subjective.

### Conclusions

When considering ADHD, internalising and externalising symptoms across childhood and adolescence, heterogeneity in individual trajectories can be usefully summarised in terms of a small number of developmental subtypes. A model with six developmental subtypes was considered optimal in this study. Subtypes included two normative subtypes (‘unaffected’ and ‘normative maturing’) and four subtypes that showed elevated mental health symptoms, three of which showed evidence of developmentally coupled symptom elevations in all three domains, and one of which was characterised by a late onset of symptoms. Covariate analyses suggested that males and bully victims tend to have complex mental health profiles; academic achievement and smoking during pregnancy have generalised associations with mental health irrespective of trajectory or combination of symptoms; and maternal post-natal depression is primarily related to symptoms that are already in evidence by childhood.

## Data Availability

Data and other relevant materials can be made available by request to the first author.

## Code availability

Code can be made available by request to the first author.
